# Skeletal Muscle Haemangioma: A Cause for Chronic Pain about the Knee: A Case Report

**DOI:** 10.1155/2012/452651

**Published:** 2012-09-09

**Authors:** Kopuri Ravi Kiran, T. V. Suresh Babu, S. Suresh Babu, K. Deepti

**Affiliations:** ^1^Department of Orthopaedics, Dr. PSIMS & RF Chinoutpalli, Gannavaram, Andhra Pradesh, Vijayawada 521101, India; ^2^Consultant Radiologist, Nagarjuna Hospital, Kanuru, Andhra Pradesh, Vijayawada 520007, India

## Abstract

Skeletal muscle haemangiomas are uncommon soft tissue tumors; more than 90% are misdiagnosed initially. They present as chronic pain and swelling in a muscle with or without a history of trauma. Plain X-rays, bone scans, computerized tomography (CT) studies, and angiography studies may not always be specific for this tumor. Diagnostic ultrasound is an appropriate initial imaging modality for suspected haemangioma, although magnetic resonance imaging is the investigation of choice. Many treatment modalities for the symptomatic haemangiomas are available of which surgical excision is the most preferred. We present an unusual case of pain, swelling, and restriction of movements in the right knee following an episode of trauma in a 12-year-old boy who was being followed for 1 year by a general practioner and later referred to us. The patient was diagnosed to have intramuscular cavernous haemangioma in the vastus medialis by us for which he was treated by surgical excision and followed for 1 year and found to have no recurrence. The clinical features, radiological picture, pathological histology, diagnostic tools, and treatment options have been discussed.

## 1. Introduction

Haemangiomas constitute 7–10% of all soft tissue tumors [1]. Intramuscular haemangioma is a rare entity accounting for 0.8% of all haemangiomas [2]. There is a general agreement that females are more commonly affected than males [3]. Intramuscular haemangiomas may present as a cause of persistent pain, swelling and may present as a perceived injury [2]. Diagnostic ultrasound is an appropriate initial imaging modality for suspected haemangioma, although magnetic resonance imaging is the investigation of choice [4]. They are of interest to the surgeon because their location may present diagnostic and therapeutic dilemma. 

Current literature reports intramuscular cavernous haemangioma of the vastus medialis in a 12-year-old boy for which surgical excision was performed. Patient was followed for 1 year and found to have no signs of recurrence.

## 2. Case Report

An 12-year-old boy was referred to us by a practioner with a swelling in the left knee region associated with pain which aggravates on running since 1 year with history of preceding trauma. There was no history of associated constitutional symptoms, bleeding tendencies, or other joint involvement. Patient was under treatment with the practioner for the past 1 year and during this period aspiration of the swelling was done thrice which revealed blood according to the patient's attendant. Throughout this period, swelling gradually increased in size inspite of aspiration.

Examination revealed an asymmetric, boggy, noncompressible, and nonpulsatile swelling in the anteromedial aspect of left suprapatellar region measuring about 4 × 3 cms. The overlying skin was normal with no discoloration and no local raise of temperature. The fossae on either side of the patellar tendon were normal and the patellar tap was negative. There was no distal neurovascular deficit or bruit. Limb length was equal to the contralateral limb. Knee movements were of full range.

With the differential diagnosis of suprapatellar bursitis, synovial dysplasia, and extra-articular soft tissue tumor, patient was subjected to further investigations.

**Figure 1:**
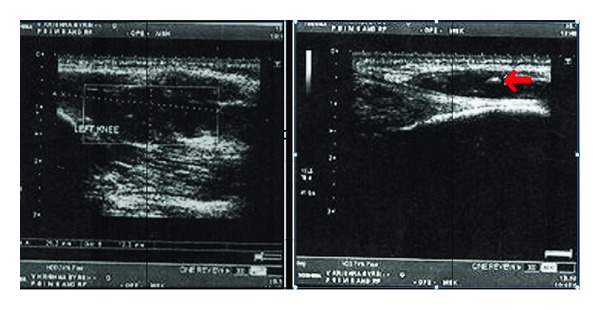
Ultrasound reveals a hypoechoic irregular mass located in the muscular plane with evidence of multiple calcific areas (marked with red arrow).

**Figure 2:**
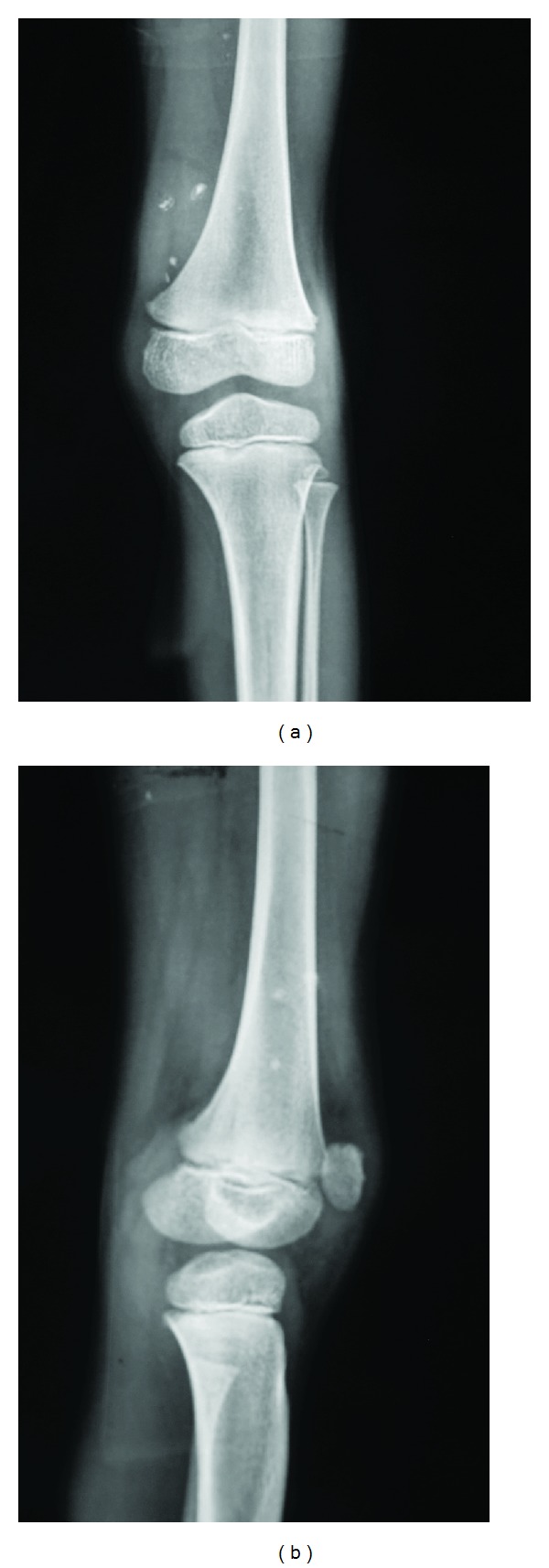
AP and lateral views of knee joint shows multiple phleboliths and minimal soft tissue swelling in suprapatellar region.

The patient had old X-rays of which the most recent was dated 5 months ago which was unremarkable except for minimal soft tissue swelling in suprapatellar region. Ultrasound revealed a hypoechoic irregular mass located in the muscular plane with evidence of increased vascularity and multiple calcific areas, all these features were favoring the diagnosis of haemangioma (Figure 1). As ultrasound revealed calcifications X-ray of the left knee was repeated which showed multiple phleboliths in addition to minimal soft tissue swelling (Figures 2(a) and 2(b)).

**Figure 3:**
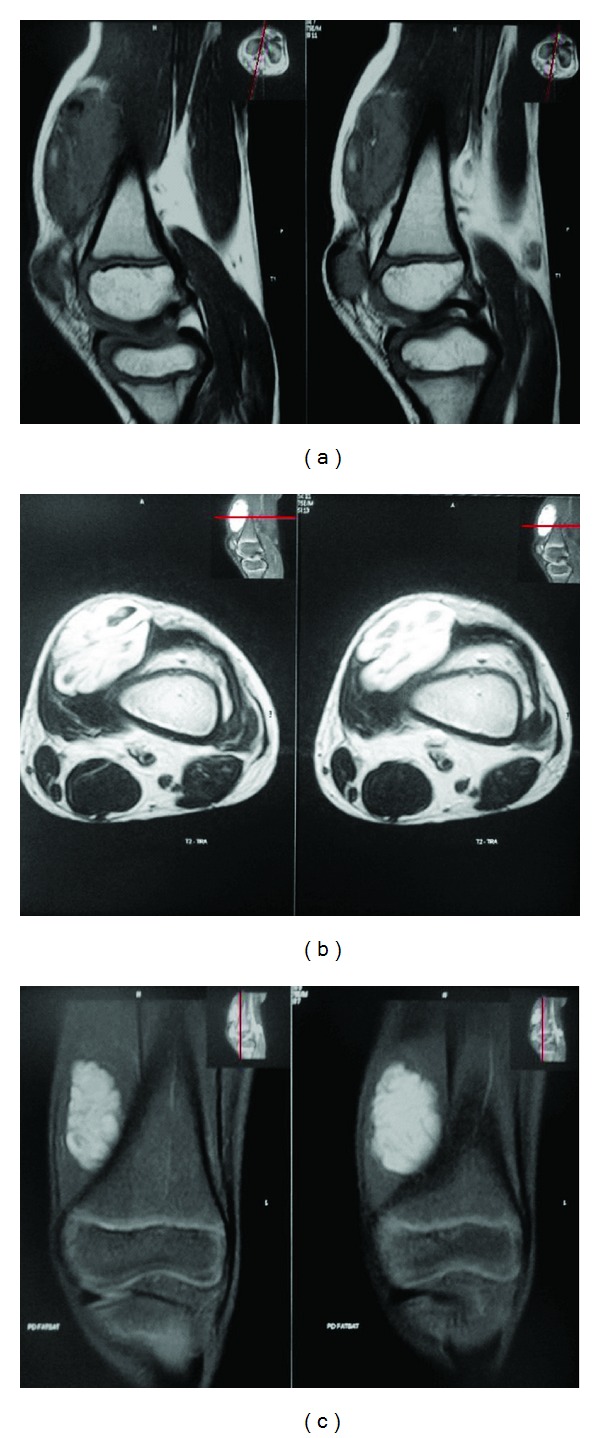
MRI reveals soft tissue mass in the vastus medialis which with high signal intensity on *T*_1_, *T*_2_, and PD FAT SAT images, with hypointense calcifications within.

Patient was planned for surgical excision and a preoperative MRI was done which revealed soft tissue mass of about 3.7 × 3.2 cms in the vastus medialis which was of high-signal intensity on *T*_1_, *T*_2_, and PD FAT SAT images, with hypointense calcifications within indicating the mass could be an intramuscular haemangioma in the vastus medialis (Figures 3(a), 3(b), and 3(c)). 

**Figure 4:**
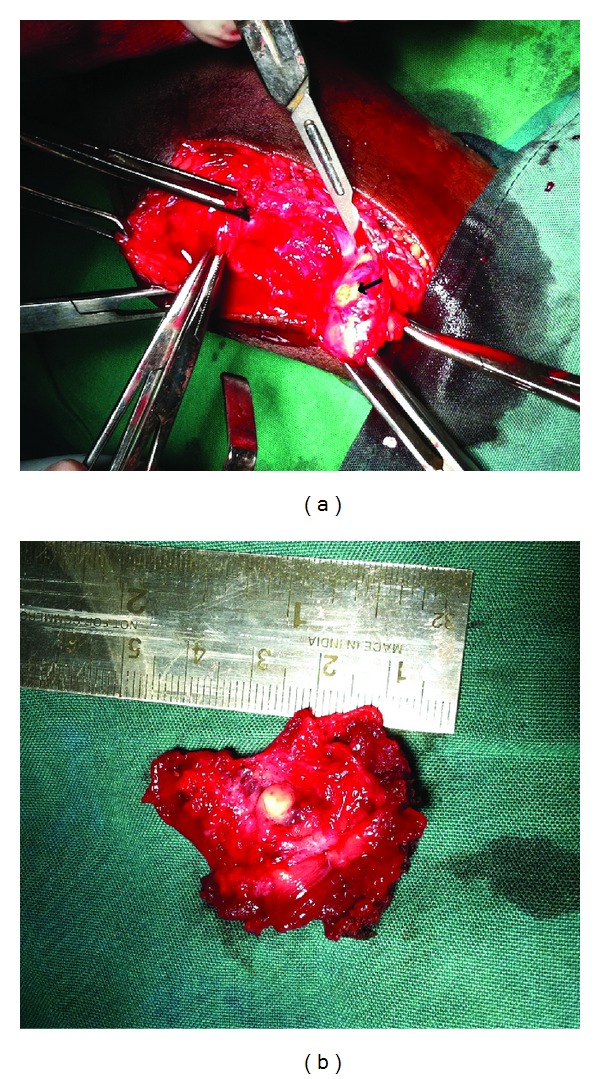
Vascular mass in the vastus medialis muscles with calcifications was encountered (calcification marked with black arrow).

At operation, a vascular mass involving the vastus medialis muscles with calcifications was encountered (Figures 4(a), and 4(b)). 

**Figure 5:**
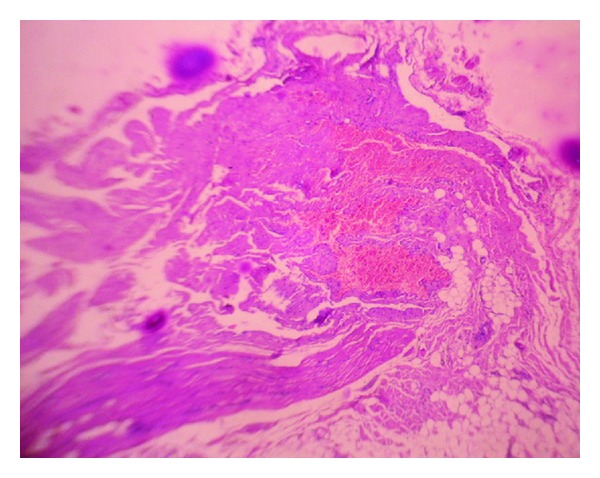
Microscopy shows skeletal muscle bundles and adipose tissue interspersed with large thick-walled blood vessels and few capillary-sized blood vessels filled with RBCs.

Microscopically, skeletal muscle bundles interspersed with lobules of thick-walled blood vessels and few capillary-sized blood vessels filled with RBCs, areas of calcification, and a peripheral rim of adipose tissue was seen. No sign of inflammation were observed. These findings were in favor of cavernous type of intramuscular haemangioma (Figure 5).

The patient had an uneventful postoperative course. He had been followed for one year and was found to be free of symptoms and showed no evidence of recurrence.

## 3. Discussion

Haemangiomas constitute 7–10% of all soft tissue tumors [1]. Intramuscular cavernous haemangiomas represent less than 1% of all haemangiomas [5]. The younger age groups are most frequently affected with 85% of cases having occurred under the age of 30 years, among which 30% are seen in lower extremities with quadriceps being the most common muscle involved [6]. There are reports that females are slightly more affected though studies also reveal no predilection [2]. There is no agreement about the etiology of these tumors, their appearance is often linked to trauma but much data exists suggesting a congenital origin [7]. More than 90% of these lesions are misdiagnosed preoperatively, as clinicians fail to consider the diagnosis because intramuscular haemangiomas are often deep seated and extremely variable in size and consistency [8]. Intramuscular haemangiomas progressively enlarge but never metastasize [9].

Clinically, haemangiomas present with swelling and pain, history of trauma is usually present in 17% of the patients [2]. A noncompressible mass without bruit is almost always present, few patients may also show bluish discoloration of overlying skin and superficial dilated veins [6]. When present either intra-articular or juxta-articular in location, haemangiomas may cause restriction of joint motion [10]. Delays or misdiagnoses are possible due to intermittent periods without pain. Scott in 1957 stated that only 8% of the haemangiomas were correctly diagnosed preoperatively [2]. Aspiration of the mass yields blood, which might be confused with hematoma in the presence of history of trauma, one quick way to differentiate haemangioma from haematoma is lighter color of the former as compared to the darker appearance of the hematoma. Moreover, the swelling does not decrease in the size after aspiration in case of haemangioma [11]. Complications of the haemangioma include functional impairment, skin necrosis of the overlying skin, bone erosion, entrapment of vessels and nerves, cardiac failure, thrombocytopenia, and consumptive coagulopathy (Kasabach-Merritt syndrome) [6, 12].

Radiographs are generally inconclusive revealing only an abnormal soft tissue mass. Occasional calcified phleboliths may be present in 25% of cases aiding in the diagnosis but does not appear to be a constant feature. Ultrasonograms may reveal complex hypoechoic mass and if phleboliths are present, acoustic shadowing may also be documented. MRI is very promising investigation, which is noninvasive, helps in diagnosing and defining the location and extent of the haemangioma though the cost and availability may be a limiting factor. Haemangiomas appear as areas of high signal intensity on both *T*_1_ and *T*_2_ images in contrast to most soft tissue tumors, which show intermediate signal intensity on *T*_1_ and high signal intensity on *T*_2_ [2, 4, 6, 13].

The term “haemangioma” is commonly misused to describe any type of vascular abnormality, including vascular malformation, in both medical and surgical fields, Hassanein et al. revealed in a study that the term was incorrectly used in 71.3% of publications on the PubMed database [14]. Haemangiomas are hamartomas or developmental anomalies in which natural cells (in contrast to neoplasms) are present in abnormal numbers. They are classified as capillary, cavernous, venous, arteriovenous, epithelioid, granulation tissue type, and miscellaneous. The vast majority are cavernous (50%), followed by capillary (25%). Cavernous haemangiomas are larger and deeper and are composed of dilated, blood-filled spaces lined by flattened endothelium. Occasionally calcification may be seen [2, 6].

Intramuscular cavernous haemangiomas do not undergo spontaneous regression; hence in symptomatic cases optimal treatment is the complete surgical excision of the tumor with a surrounding margin of the normal muscle [5–7]. The 17–20% of local recurrence rate reported in the literature which is due to inadequate primary surgical excision rather than histological subtype [15]. Compression sclerotherapy using sclerosing agents [6], radiotherapy [12], embolization [12], and laser ablation [16] has also been suggested as treatment options in large diffuse lesions where surgical excision is impractical.

## 4. Conclusion

Lesions arising in muscle may create significant diagnostic uncertainty, as on clinical examination they are little different from soft tissue neoplasms and often imaging features may not be fully appreciated or easy to interpret. Skeletal muscle haemangiomas are uncommon soft tissue tumors that are completely treatable, the knowledge of their natural history, clinical findings, and imaging appearances are of great importance for proper diagnosis. These lesions are often diagnosed late or misdiagnosed as abscess, lipoma, sarcoma, fibroma, muscle hernia, dermoid cyst, lymphatic glands, or even synovitis when presenting juxta articular.

The aim of presenting this case is to emphasize that, in cases with chronic or recurrent painful swelling in a muscle or adjacent to a joint with or without an episode of trauma especially in a young individual should alert the surgeon to this diagnosis. Investigations such as ultrasound can diagnose the lesion but may not be able to delineate its extent, MRI is preferred for identifying the exact location, extent, and size of the lesion for planning surgical excision, which is the most preferred treatment for these lesions.
